# Agricultural insurance and rural revitalization—an empirical analysis based on China’s provincial panel data

**DOI:** 10.3389/fpubh.2023.1291476

**Published:** 2023-12-04

**Authors:** Chao Zhou, Jia Liu, Shenwei Wan, Hongling Zheng, Song Chen

**Affiliations:** ^1^Research Center of the Economic and Social Development of Henan East Provincial Joint, Shangqiu Normal University, Shangqiu, China; ^2^School of Economics and Management, Northeast Forestry University, Harbin, China; ^3^School of Agricultural Economics and Rural Development, Renmin University of China, Beijing, China; ^4^School of Ocean, Tangshan Normal University, Tangshan, China; ^5^College of Marxism, Yunnan Agricultural University, Kunming, China

**Keywords:** rural revitalization, TOPSIS entropy weight method, agricultural insurance, GMM, threshold model

## Abstract

Agricultural insurance is a kind of compensation insurance designed to provide protection for the economic losses caused by insured accidents suffered by agricultural producers in agricultural production. Rural revitalization refers to the strategy of improving the level of rural economic, social and cultural development and achieving coordinated and sustainable development of urban and rural development. Agricultural insurance can effectively diversify risks and reduce losses for agricultural producers, which plays an important role in stabilizing farmers’ income, helping rural economic development, and promoting rural revitalization. Based on the theoretical analysis of the mechanism of agricultural insurance on rural revitalization, this paper empirically studies the effect of agricultural insurance on rural revitalization by using panel data from various provinces in China from 2011 to 2020.^1^ In this paper, the TOPSIS entropy weight method, the system generalized method of moments (GMM) and the threshold model are used to calculate the actual development level of rural revitalization in each province of China, the promotion effect of agricultural insurance on the development level of rural revitalization and the promotion of rural revitalization in five dimensions, and whether there is a threshold effect of agricultural insurance on rural revitalization. The empirical results show that: (1) The level of rural revitalization in various provinces in China shows a dynamic trend of “overall slow rise, with obvious differences between provinces.” (2) Improving the development level of agricultural insurance can drive the improvement of China’s rural revitalization level, and every 1 unit increase in the development level of agricultural insurance will drive the level of China’s rural revitalization to increase by 0.1633 units. At the same time, the role of agricultural insurance on social etiquette and civility is not significant, and the role of the remaining four rural revitalization goals is significant. (3) Agricultural insurance has a significant effect on the level of rural revitalization in eastern provinces, but does not play a significant role in rural revitalization in central and western provinces. (4) The role of agricultural insurance on rural revitalization has a double threshold effect. Accordingly, this paper puts forward some suggestions for increasing the capital investment in agricultural insurance, innovating the new mode of agricultural insurance operation, promoting the in-depth development of agricultural insurance according to local conditions, and reasonably adjusting the capital investment of agricultural insurance. Finally, because the data used in this paper do not cover the entire process of rural revitalization and the research is mainly carried out from a macro perspective, there are still some shortcomings in this paper.

## 1 Introduction

Agriculture is the foundation of a country and the foundation of a strong country. Agricultural development is related to the prosperity and stability of the country and the well-being of people’s livelihood, and agriculture is a basic industry that provides support for the construction and development of the national economy. Whether or not agricultural development is fast, stable, and good directly reflects whether a country has the objective conditions for long-term stable development. The law of modern social development shows that with the continuous improvement of the level of agricultural productivity, traditional agricultural agriculture has begun to gradually transform into modern agriculture, and has become an agriculture that widely applies modern science and technology, provides means of production for modern industry, and applies modern production management methods. This is the universal law for the vast majority of countries in the world to realize the transformation from a large agricultural country to an agricultural power, and it is also the core task of contemporary China’s agricultural and rural modernization.

China, a developing country with thousands of years of farming history, has long maintained a traditional agricultural development model in many rural areas. Most of China’s agricultural producers still maintain traditional planting habits, and always attribute natural and man-made disasters encountered in agricultural production to bad luck, and rarely think of avoiding risks through agricultural insurance (1). However, this situation is quietly changing with the vigorous development of China’s rural revitalization (agricultural and rural modernization). The strong endogenous demand for agricultural insurance for rural revitalization has also made agricultural insurance develop by leaps and bounds in the past two decades. China Agricultural Insurance was born in 2004, agricultural insurance is the insured (usually agricultural producer) to pay a certain amount of premiums to the insurer (usually the insurance company), the insurer within the period specified in the agricultural insurance to compensate for the loss of the subject matter of the insurance. China’s agricultural insurance is mainly divided into four categories: weather index insurance (2), yield insurance, price insurance and income insurance (3). As a risk management tool and resource allocation tool, the role of agricultural insurance in rural revitalization has attracted increasing attention.

Agricultural insurance is a common practice in countries with a market economy to support agricultural development. At present, the status of agricultural insurance in China’s economic and social development has attracted more and more attention. Agricultural insurance can effectively disperse and transfer farmers’ property risks, reduce the impact of natural disasters on agricultural production, stabilize farmers’ incomes, and promote the development of agriculture and rural economy. Rural revitalization is an important measure to solve the problems of China’s agriculture, rural areas and farmers, and to realize the modernization of agriculture and rural areas. It can be seen that agricultural insurance can promote rural revitalization, and rural revitalization requires the sustainable and healthy development of agricultural insurance. Agricultural insurance can provide agricultural risk diversification mechanism and financial support for agricultural construction for the full implementation of the rural revitalization strategy (4). Specifically, on the one hand, agricultural insurance realizes the timely dispersion of agricultural risk losses in time and space through the large number theorem, which effectively improves the ability of agricultural risk management and control, thereby ensuring the stability of agricultural producers’ income and reproduction investment, and providing a strong economic guarantee for agricultural production. On the other hand, agricultural insurance has completed the preliminary work of crop value assessment, quantified agricultural products that are difficult to measure into calculable collateral, indirectly helped agricultural producers open up the last channel of agricultural credit, and increased the opportunities for agricultural operators to obtain credit financing. Since the beginning of the new century, China has successively issued 20 Central Document No. 1 on guiding the development of agricultural insurance, fully affirming the importance of agricultural insurance to China’s rural revitalization.

In the context of rural revitalization, agricultural insurance is no longer limited to ensuring the safety of agricultural production, and is more endowed with the function of assisting rural revitalization.^2^ At present, most of the research on agricultural insurance and rural revitalization focuses on the four aspects of rural economy, farmers’ income, poverty reduction effect and agricultural total factor productivity (5, 6), and there are certain differences in the research conclusions. Most scholars basically affirm the poverty reduction and income increase effect of agricultural insurance (7), and the positive effect of agricultural insurance on improving agricultural total factor productivity (8). But there are studies that come to different conclusions. Some scholars believe that agricultural insurance premium expenditure is a substitute for total income, so agricultural insurance can only guarantee farmers’ operating income, but cannot guarantee total income (9). Some scholars have pointed out that the high proportion of premium subsidy policies has caused moral hazard and adverse selection of farmers, and insurance participation has a negative inhibitory effect on the allocation of agricultural production factors and production efficiency (10, 11). In addition, scholars Hou and DA discussed the impact mechanism of agricultural insurance on agricultural green development and found that agricultural insurance has a significant inhibitory effect on agricultural green development (12). These research results lay a solid foundation for the research of this paper.

Theory and practice show that in-depth study and promotion of China’s agricultural insurance and continuous meeting of farmers’ agricultural insurance needs are the focus of breakthroughs in rural revitalization in the future. Compared with the existing literature (13–15), this paper will systematically explore the dynamic impact of China’s agricultural insurance on the level of rural revitalization, rather than just limiting the perspective to a certain aspect of rural revitalization.

The research framework of this paper is as follows: First, based on the research results in related fields at home and abroad, the mechanism of agricultural insurance on rural revitalization is theoretically analyzed. Specifically, it analyzes the industry-driven effect, ecological improvement effect, civilization and education effect, normative governance effect and poverty reduction and income-increasing effect corresponding to the five goals of rural revitalization: “thriving businesses, pleasant living environments, social etiquette and civility, effective governance, and prosperity” (16). Secondly, based on the data of various provinces in China from 2011 to 2020, an empirical analysis is carried out on the role of agricultural insurance in rural revitalization. Specifically, the first step is to use the TOPSIS entropy weight method to measure and rank the level of rural revitalization in various provinces in China, and analyze the results. In the second step, the system generalized method of moments (GMM) method is used to study the relationship between agricultural insurance and the rural revitalization level of Chinese provinces and its five dimensions, and the results are analyzed. In the third step, China’s provinces are divided into three regions according to their economic development level and geographical location: eastern, central and western regions, and the regional heterogeneity of the impact of agricultural insurance on rural revitalization is explored in depth and the results are analyzed. The fourth step is to study whether agricultural insurance has a threshold effect on rural revitalization and analyze the results. Finally, the research conclusions are drawn and the policy suggestions for better play the positive role of agricultural insurance to promote rural revitalization are put forward. At the same time, this paper also points out the existing shortcomings and future research directions and priorities.

## 2 Mechanism of action

After 20 years of rapid development, the quality of agricultural insurance in China has been significantly improved, the guarantee and service functions of agricultural insurance for rural revitalization have been effectively played (17), and increasing the depth and density of agricultural insurance can make positive contributions to the development of rural revitalization (18).

**Figure 1:**
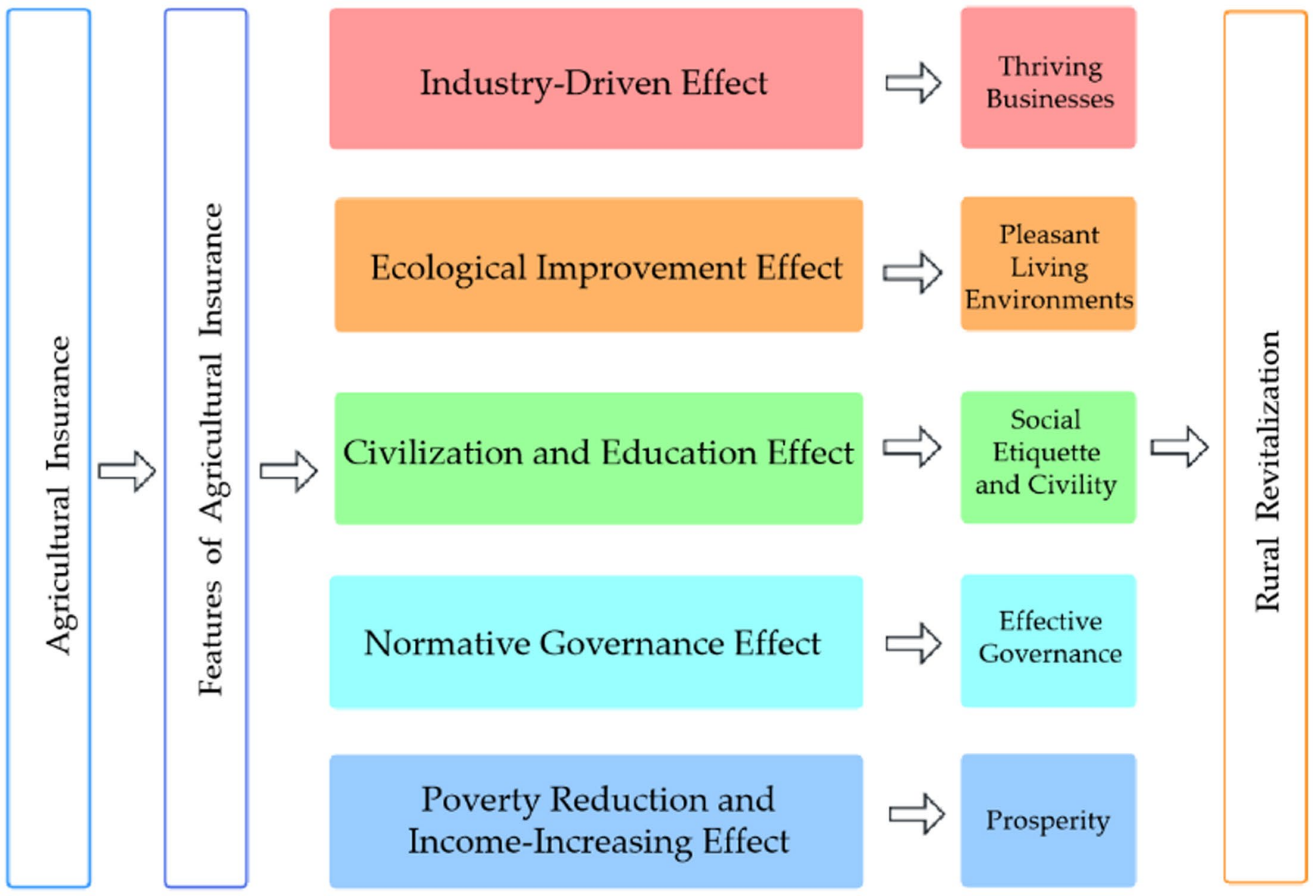
Mechanisms of agricultural insurance for rural revitalization.

Based on the five aspects of rural revitalization, this paper divides the mechanism of agricultural insurance on rural revitalization into industry-driven effect, ecological improvement effect, civilization and education effect, normative governance effect and poverty reduction and income-increasing effect (19). The mechanism of action is shown in Figure 1.

### 2.1 Industry-driven effect

To achieve rural revitalization, thriving businesses is the top priority. The prosperity of industry is the material basis for realizing rural revitalization and the foundation and premise for solving all problems in rural areas. A large number of empirical studies have shown that agricultural insurance is not only a “stabilizer” to ensure food production, but also a “booster” for the development of characteristic agricultural industries. First, agricultural insurance is an important tool for agricultural producers to prevent production and operation risks and stabilize income expectations, which is conducive to stabilizing the development of the agricultural industry and ensuring food security (20). Second, the regional, decentralized and difficult large-scale operation of characteristic agriculture determines that farmers will inevitably face greater risks in the process of its development, and agricultural insurance provides risk management for the development of characteristic agriculture, which is conducive to promoting the development of characteristic industries in rural areas (21).

### 2.2 Ecological improvement effect

To achieve rural revitalization, pleasant living environments is the key point. Most of China’s rural areas are remote, information blocked, and economically backward, and agricultural producers’ first pursuit of agriculture is often to increase production and income rather than a good ecological environment, which has largely contributed to the deterioration of the rural environment (22). The “agricultural insurance + ecological agriculture” mechanism can solve this problem well. First, agricultural insurance incentivizes agricultural producers to change traditional agricultural production methods (23). Compared with traditional agriculture, ecological agriculture pays more attention to the green and pollution-free production. The “agricultural insurance + ecological agriculture” mechanism can prevent the risk of reducing the yield due to the reduction of chemical fertilizer and pesticide application through agricultural insurance, guide agricultural producers to change traditional agricultural production methods, and effectively curb pollutant emissions (24). Second, agricultural insurance adjusts the allocation of resources among agricultural products. The development of agricultural insurance will affect the resource allocation of agricultural producers between different crops, guide producers to adjust crop structure, and reduce the emission of fertilizer pollutants (25, 26).

### 2.3 Civilization and education effect

To achieve rural revitalization, social etiquette and civility are the guarantee. In the context of rural revitalization, the function of agricultural insurance has broken through the narrow scope of agricultural production and extended to agriculture, rural areas, and farmers. Part of the agricultural insurance funds invested in rural areas has led to the construction of transportation, education and other infrastructure, to a certain extent, changing the status quo of isolation and backwardness in China’s rural areas, and laying a solid foundation for rural residents to improve their quality. In addition, in order to smoothly provide insurance services in rural areas, insurance companies will also provide a series of agricultural technical guidance and training to agricultural producers to help them improve their production technology and management level.

### 2.4 Normative governance effect

To achieve rural revitalization, effective governance is the foundation. In recent years, China’s agricultural insurance is gradually expanding the scope of protection and expanding insurance liability, and the mode of agricultural insurance is changing from single compensation to whole-process agricultural risk management, from “small agricultural insurance” to “large agricultural insurance.” For example, Jiangsu Province and other regions in China have incorporated agricultural insurance into local disaster prevention and mitigation plans, mobilized agricultural producers to cooperate with government departments in disaster prevention and mitigation work, and written agricultural insurance into village regulations and people’s agreements, so as to improve farmers’ awareness of disaster prevention and mitigation and their ability to resist risks (27). Insurance companies cooperate with municipal (county) agricultural and rural bureaus, town agricultural and rural offices and other government departments to hold agricultural insurance publicity to bring relevant economic and legal knowledge, which is also conducive to the improvement of rural governance capabilities.

### 2.5 Poverty reduction and income-increasing effect

To achieve rural revitalization, prosperity is fundamental. The study found that agricultural insurance plays a huge role in long-term poverty reduction and increasing production and income. First, agricultural insurance promotes high-quality agricultural development by improving agricultural total factor productivity, and improves the endogenous ability of agriculture to cope with risks and high-quality development (28), thus establishing a long-term poverty reduction mechanism. Second, agricultural insurance improves the financing capacity of agricultural producers by providing credit enhancement guarantees. Agricultural insurance can help agricultural producers stabilize income expectations, improve repayment ability, and indirectly improve farmers’ financing ability, thereby increasing the working capital in the hands of agricultural producers, motivating them to increase investment and expand production, so as to increase production and income (29).

## 3 Empirical research and analysis of results

### 3.1 Dynamic measurement of China’s rural revitalization level

#### 3.1.1 Date and methods

In 2010, the “No. 1 Document” of the central government proposed the strategy of “increasing the premium subsidy of the central government to the central and western regions, and encouraging all localities to subsidize the premiums of insurance for characteristic agriculture and farmhouses.” In view of this, this paper selects panel data of indicators related to rural revitalization in various provinces in China from 2011 to 2020, and all the original data are derived from official statistical data such as *China Statistical Yearbook*, *China Rural Statistical Yearbook* and Statistical Yearbook of Chinese provinces, and the missing values are supplemented by linear interpolation.

Based on the obtained data, refer to the existing research results (16, 30), using the TOPSIS entropy weight method to measure the rural revitalization index system, the specific calculation steps are as follows:

Step 1: Standardize the measurement data of each indicator in the evaluation system using the range method to eliminate differences in the order of magnitude and dimension of each indicator. As the selected indicators are all positive indicators, the specific processing method for this step is:


(1) $$Y_{ij}=\frac{X_{ij}-m_{ij}}{M_{ij}-m_{ij}}+0.00001$$

In Formula (1): i represents the region, j represents the evaluation index (*i* = 1, 2, …, *m*; *j* = 1, 2, …, *n*), *m* is the number of evaluation objects, and *n* is the number of indicators. $X_{ij}$ and $Y_{ij}$ represents the raw and dimensionless indicator data, $M_{ij}$ and $m_{ij}$ represents the maximum and minimum values, respectively.

Step 2: Calculate the entropy value $E_{j}$ of each indicator $Y_{ij}$ :


(2) $$E_{j}=-\frac{1}{\ln\left(m\right)}\overset{m}{\underset{i=1}{\sum }}\left[\left(\frac{Y_{ij}}{\sum_{i=1}^{m}Y_{ij}}\right)\ln\left(\frac{Y_{ij}}{\sum_{i=1}^{m}Y_{ij}}\right)\right]$$

Step 3: Empower the indicator $Y_{ij}$ and find the weight $W_{j}$ :


(3) $$W_{j}=\frac{1-E_{j}}{\sum_{i=1}^{n}\left(1-E_{j}\right)}$$

Step 4: Build the weighted matrix R.


(4) $$R={\left(r_{\mathrm{ij}}\right)}_{n\times m}$$

In Formula (4): $r_{\mathrm{ij}}=W_{j}\times Y_{ij}$ .

Step 5: Determine the ideal solution $Z_{j}^{+}$ and the inverse ideal solution $Z_{j}^{-}$ by the weighted matrix R established in step 4:


(5) $$\;Z_{j}^{+}=\left(\max r_{i1},\max r_{i2},\ldots \max r_{in}\right)$$

(6) $$Z_{j}^{-}=\left(\min r_{i1},\min r_{i2},\ldots \min r_{in}\right)$$

Step 6: Calculate the distance $d_{i}^{+}$ and $d_{i}^{-}$ from each measurement scheme to the ideal solutions $Z_{j}^{+}$ and the inverse ideal solution $Z_{j}^{-}$ :


(7) $$d_{i}^{+}=\sqrt{\overset{n}{\underset{j=1}{\sum }}{\left(Z_{j}^{+}-r_{\mathrm{ij}}\right)}^{2}}$$

(8) $$d_{i}^{-}=\sqrt{\overset{n}{\underset{j=1}{\sum }}{\left(Z_{j}^{-}-r_{\mathrm{ij}}\right)}^{2}}$$

Step 7: Calculate $C_{i}\;$ which is the similarity of each assessment solution to the ideal solution:


(9) $$C_{i}=\frac{d_{i}^{-}}{d_{i}^{+}+d_{i}^{-}}$$

It can be seen that the value range of the $C_{i}\;$ is [0,1]. The larger the $\;C_{i}$ , the higher the level of rural revitalization in the region; Conversely, the lower the level of rural revitalization in the region.

#### 3.1.2 Construction of rural revitalization level measurement system

China’s rural revitalization focuses on coordinated development. To measure the level of China’s rural revitalization, it is necessary to consider the thriving businesses, pleasant living environments, social etiquette and civility, effective governance, and prosperity.

This paper starts from the five dimensions of the general requirements of the rural revitalization strategy, with reference Zeng et al. and other scholars’ research results (31), selected 16 positive indicators such as “Value added of primary industry (billion yuan)” to build a measurement index system for the development level of rural revitalization, as shown in Table 1.

**Table 1:** Indicator system for measuring the development level of rural revitalization.

Dimension	Level 1 indicators	Secondary indicators
Rural revitalization	Thriving businesses	Value added of primary industry (billion yuan)
		Gross regional product (billion yuan)
		Total investment in fixed assets (billion yuan)
		Total power of agricultural machinery (million kilowatts)
	Pleasant living environments	Forest cover (%)
		Non-hazardous treatment rate of domestic waste (%)
		Integrated production capacity for water supply (10,000 m^3^/day)
		Number of beds in health institutions (10,000 beds)
	Social Etiquette and civility	Integrated population coverage of television programs (%)
		Local financial expenditure on education (billion yuan)
		Number of institutions of comprehensive cultural stations in townships (units)
	Effective governance	Number of village council units (units)
		General public service expenditures of local finances (billion yuan)
		Number of legal entities of public administration, social security and social organizations (units)
	Prosperity	Disposable income per agricultural producer (yuan)
		*Per capita* cash consumption expenditure on culture, education and recreation of rural residents (yuan)

#### 3.1.3 Measurement results and analysis of rural revitalization level

**Figure 2:**
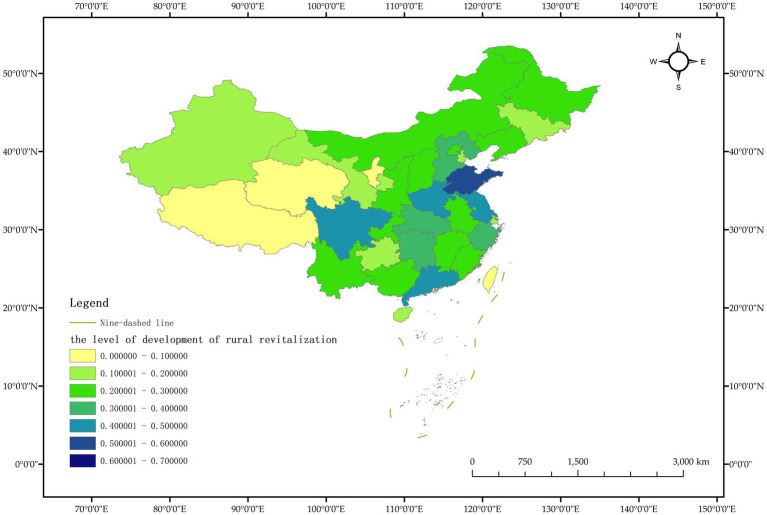
Schematic representation of the level of rural revitalization by province in China in 2011.

**Figure 3:**
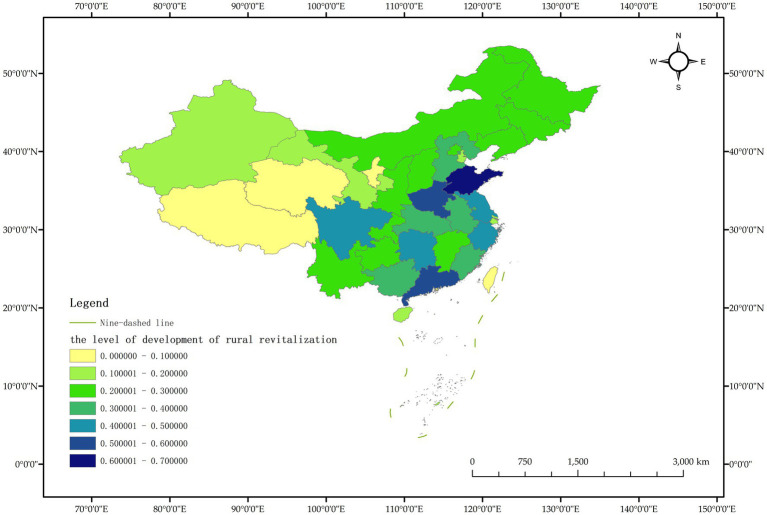
Schematic diagram of the level of rural revitalization by province in China in 2015.

**Figure 4:**
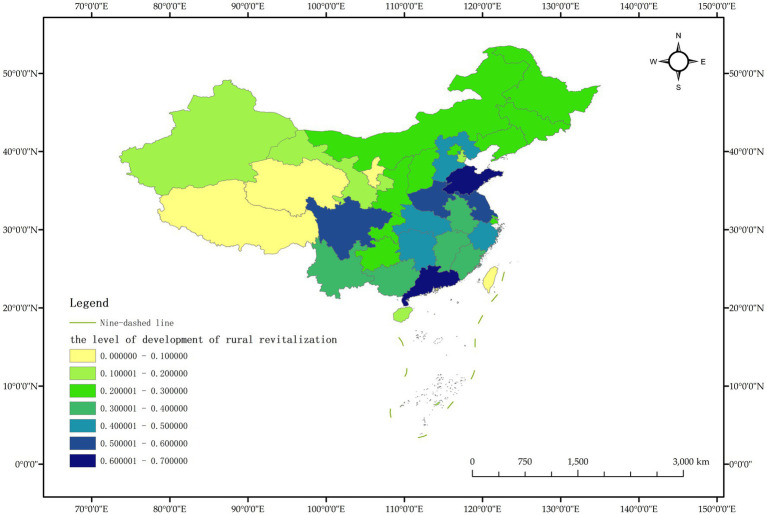
Schematic diagram of the level of rural revitalization by province in China in 2020.

As a commonly used comprehensive evaluation method, the TOPSIS entropy weight method, also known as the approximate ideal solution ranking method, can make full use of the relevant information of the original data in the sample group to obtain the distance between each evaluation scheme and the ideal scheme, and then assign weight to each index and calculate the comprehensive evaluation index. According to the index data in the measurement index system of rural revitalization development level (Table 1), the TOPSIS entropy weight method was used to measure the rural revitalization level of Chinese provinces, and the dynamic measurement values of rural revitalization development level of Chinese provinces from 2011 to 2020 were obtained, and ranked according to the dynamic measurement values of rural revitalization development level of Chinese provinces in 2020 from high to low, as shown in Table 2. In order to observe the dynamic changes of rural revitalization level of Chinese provinces more clearly, the data of Chinese provinces in 2011, 2015 and 2020 were selected for visualization, and the results are shown in Figures 2–4.

**Table 2:** The dynamic measurement values of rural revitalization development level of Chinese provinces from 2011 to 2020.

	2011	2012	2013	2014	2015	2016	2017	2018	2019	2020	Rankings for 2020
Shandong	0.563	0.586	0.594	0.612	0.620	0.611	0.624	0.622	0.628	0.628	1
Guangdong	0.453	0.476	0.482	0.504	0.524	0.545	0.560	0.573	0.615	0.615	2
Henan	0.448	0.472	0.484	0.506	0.513	0.512	0.525	0.542	0.567	0.567	3
Jiangsu	0.454	0.469	0.457	0.483	0.495	0.515	0.516	0.535	0.564	0.564	4
Sichuan	0.445	0.463	0.466	0.486	0.495	0.508	0.525	0.541	0.531	0.531	5
Zhejiang	0.399	0.410	0.399	0.414	0.424	0.435	0.440	0.449	0.464	0.464	6
Hunan	0.393	0.410	0.411	0.431	0.439	0.429	0.440	0.448	0.463	0.463	7
Hubei	0.324	0.345	0.350	0.373	0.383	0.394	0.401	0.406	0.418	0.418	8
Hebei	0.361	0.370	0.363	0.380	0.380	0.383	0.395	0.416	0.412	0.412	9
Anhui	0.291	0.306	0.309	0.324	0.327	0.334	0.333	0.351	0.360	0.360	10
Guangxi	0.267	0.279	0.277	0.295	0.302	0.309	0.314	0.329	0.337	0.337	11
Fujian	0.286	0.295	0.293	0.307	0.309	0.314	0.321	0.330	0.335	0.335	12
Jiangxi	0.284	0.293	0.275	0.292	0.297	0.303	0.312	0.325	0.331	0.331	13
Yunan	0.230	0.247	0.249	0.267	0.270	0.283	0.296	0.310	0.320	0.320	14
Shaanxi	0.265	0.275	0.273	0.285	0.277	0.278	0.280	0.289	0.295	0.295	15
Heilongjiang	0.237	0.253	0.250	0.262	0.269	0.277	0.278	0.286	0.294	0.294	16
Liaoning	0.285	0.293	0.281	0.295	0.284	0.276	0.272	0.283	0.291	0.291	17
Guizhou	0.191	0.205	0.207	0.231	0.238	0.241	0.249	0.263	0.274	0.274	18
Beijing	0.239	0.240	0.235	0.255	0.249	0.263	0.257	0.293	0.242	0.242	19
Inner Mongolia	0.201	0.210	0.206	0.225	0.222	0.223	0.225	0.230	0.235	0.235	20
Chongqing	0.201	0.202	0.195	0.216	0.216	0.224	0.227	0.236	0.235	0.235	21
Jilin	0.196	0.204	0.200	0.211	0.217	0.220	0.218	0.226	0.228	0.228	22
Shanxi	0.227	0.235	0.229	0.245	0.245	0.238	0.228	0.230	0.224	0.224	23
Shanghai	0.189	0.194	0.175	0.196	0.195	0.201	0.203	0.217	0.223	0.223	24
Xinjiang	0.140	0.147	0.148	0.162	0.165	0.172	0.183	0.190	0.198	0.198	25
Gansu	0.137	0.156	0.159	0.177	0.181	0.181	0.184	0.194	0.195	0.195	26
Hainan	0.124	0.127	0.122	0.133	0.132	0.136	0.144	0.153	0.152	0.152	27
Tianjin	0.146	0.147	0.133	0.151	0.148	0.151	0.148	0.149	0.144	0.144	28
Xizang	0.050	0.057	0.065	0.080	0.080	0.086	0.090	0.098	0.099	0.099	29
Ningxia	0.084	0.082	0.082	0.096	0.090	0.093	0.094	0.099	0.093	0.093	30
Qinghai	0.074	0.074	0.065	0.081	0.079	0.085	0.082	0.089	0.084	0.084	31

The measurement results show that the level of rural revitalization in various provinces in China shows a dynamic trend of “overall slow rise, with obvious differences between provinces.” In 2020, compared with 2011, only three provinces, Guangdong, Henan and Jiangsu, improved their rural revitalization level by more than 0.1, indicating that China’s rural revitalization progress is relatively slow overall. The largest increase in rural revitalization was in Guangdong Province, from 0.453 in 2011 to 0.615 in 2020, a total increase of 0.162; the smallest increase in rural revitalization was in Shanxi Province, which decreased from 0.227 in 2011 to 0.224 in 2020, showing negative growth. Negative growth was also recorded in Tianjin. Taking 2020 as an example, only 5 provinces had a rural revitalization level of more than 0.5, accounting for 16.13% of all provinces. The level of rural revitalization in most provinces is less than half, and the level of rural revitalization in provinces such as Tibet, Qinghai and Ningxia are less than 0.1, and the level of rural revitalization in China’s provinces varies significantly.

It is worth noting that the ranking of rural revitalization levels in economically developed areas such as Beijing, Shanghai and Tianjin in 2020 is not in the forefront, which is inconsistent with the existing research results (32). The main reason is that the five-dimensional index system of rural revitalization designed in this paper is relatively richer, in addition to the industrial development level index representing thriving businesses, it is mainly based on the five dimensions of rural revitalization and constructs the five-dimensional index representing agricultural and rural development, that is, the selected indicators focus on the comprehensive development of the five goals of rural revitalization, and do not take the economic level as the main indicator to measure the level of rural revitalization.

### 3.2 Construction of the system generalized method of moments model and analysis of empirical results

#### 3.2.1 Construction of the system generalized method of moments model

In view of the dynamic volatility and coherence of the fluctuation level of rural revitalization, the system generalized method of moments can better alleviate the endogenous problem caused by it. The system generalized method of moments is an estimation method for dynamic panel data models, which can well eliminate the bias caused by individual differences in traditional OLS (least squares) estimation, eliminate the fixed effect, and improve the consistency and validity of the model. In order to study the mechanism of agricultural insurance on the level of rural revitalization in various provinces of China and its five dimensions, and to test whether the explanatory variables lagging in the first period affect the explanatory variables in the current period, the model is established as follows:


(10) $$Y_{it}=\alpha_{0}+\alpha_{1}Y_{it-1}+\alpha_{2}AI_{it}+\beta_{1}\sum \theta CV_{it}+\varepsilon_{it}$$

In Equation (10), $Y_{it}$ is the explanatory variable, representing the overall level of development of rural revitalization and its five dimensions; $Y_{it-1}$ represents the level of development of the explanatory variable lagging by one period; $AI_{it}\;$ it is the core explanatory variable, indicating the level of agricultural insurance development; $CV_{it}\;$ it represents a vector of control variables; $\alpha_{0},\;\;\alpha_{1},\;\;\alpha_{2},\;\;\beta_{1}$ and θ are all parameters to be estimated; $\varepsilon_{it}$ is a random perturbation term, with subscript i for each region and t for year.

Since this paper uses panel data from 2011 to 2020 in various provinces in China, the dynamic panel system generalized method of moments (GMM) is used for regression analysis to eliminate the endogenous and weak instrumental variable problems of static models and dynamic differential GMM models at the same time, and obtain more accurate and scientific estimation results.

#### 3.2.2 Variable and data source

Interpreted variables: the development level of rural revitalization and the development level of its five target dimensions, the so-called five target dimensions are thriving businesses, pleasant living environments, social etiquette and civility, effective governance, and prosperity.

Core explanatory variables: the development level of agricultural insurance, the agricultural insurance loss ratio is selected to measure the development level of agricultural insurance, the main calculation method is the ratio of agricultural insurance expenditure to agricultural insurance income in each province, and also includes the economic meaning of China’s agricultural insurance market expenditure and income.

Tool variable: The proportion of agricultural insurance scale is selected as the tool variable, and the index value is the proportion of agricultural insurance expenditure in the total insurance expenditure of each province. The larger the scale of agricultural insurance, the higher the agricultural insurance expenditure, and when the value of agricultural insurance income is certain, the agricultural insurance compensation rate will be increased, and then the development level of agricultural insurance will be improved. Therefore, the variable satisfies the correlation condition of the selection of the instrumental variable, and does not directly affect the level of rural revitalization, that is, it meets the exogenous condition of the selection of the instrumental variable.

Control variables: *per capita* disposable income, mulch film coverage area, total power of agricultural machinery, rural electricity consumption, agricultural fertilizer application and grain sowing area were selected. The data of each control variable were obtained from the website of the National Bureau of Statistics and *the China Statistical Yearbook*, and the missing parts were obtained and filled by linear imputation.

A descriptive statistical analysis of each variable is shown in Table 3.

**Table 3:** Descriptive statistical analysis.

Indicator name	Sample size	Mean	Standard deviation	Min	Max
Level of development of Rural Revitalization (RR)	310	0.3088	0.1685	0.0499	1.3610
Level of development of Thriving Industry (TI)	310	0.0860	0.0514	0.0128	0.2430
Level of development of Ecologically Livable (EL)	310	0.0946	0.0377	0.0193	0.2128
Level of development of Civilization of Village Morals (CV)	310	0.0293	0.0189	0.0019	0.0976
Level of development of Effective Governance (EG)	310	0.0722	0.0907	0.0001	0.9283
Level of development of Prosperous (PP)	310	0.0266	0.0154	0.0020	0.0909
Agricultural insurance payout ratio (AI)	310	0.6375	0.2095	0.1424	1.5787
Percentage of agricultural insurance size (PA)	310	0.0840	0.0826	0.0049	0.4265
*Per capita* disposable income (PD)	310	9.3563	0.4010	8.3612	10.4606
Mulch film coverage area (MF)	310	12.4912	1.5134	7.6525	15.1494
Total power of agricultural machinery (TP)	310	7.6373	1.1255	4.5433	9.4995
Rural electricity consumption (RE)	310	4.7276	1.5329	0.1054	7.6064
Agricultural fertilizer application (AF)	310	4.7225	1.2541	1.4816	6.5738
Grain sowing area (GS)	310	7.6550	1.3117	3.8395	9.5776

#### 3.2.3 Empirical research and analysis of results

In order to verify whether the results of the system generalized method of moments (GMM) are valid, the second-order sequence correlation AR (2) and Sargan test methods are selected to test the model, aiming to distinguish the applicability of the tool variables and the accuracy of the research results. AR (2) test is mainly used to measure whether there is a sequence correlation in the residuals of the model estimation results, and the model is valid if there is no sequence correlation. The Sargan test is used to verify the overall validity of the instrumental variables selected in the system generalized method of moments (GMM). Table 4 reports the empirical results obtained by the model when the level of rural revitalization and its five dimensions are explanatory variables. The results of AR (2) test and Sargan test in the six models showed that the model had good validity and robustness.

**Table 4:** The system GMM model empirical results.

Explanatory variable	RR	TI	EL	CV	EG	PP
	(1)	(2)	(3)	(4)	(5)	(6)
L.RR	0.1632^*^ (1.73)	−0.0227^*^ (−1.76)	0.0274^***^ (3.10)	0.0308^**^ (1.98)	0.0872 (0.91)	0.0216^***^ (7.66)
AI	0.1633^***^ (2.74)	0.0152^**^ (2.31)	0.0209^***^ (2.78)	0.0025 (0.71)	0.1164^**^ (2.20)	0.0090^**^ (2.37)
PD	0.0782^***^ (3.06)	−0.0028 (−0.61)	0.0104^***^ (2.78)	0.0084^***^ (4.36)	0.0510^**^ (2.50)	−0.0060 (−1.53)
MF	−0.0115^**^ (−2.08)	−0.0002 (−0.32)	−0.0011^**^ (−2.58)	−0.0004 (−0.80)	−0.0115^**^ (−2.09)	0.0014^***^ (3.52)
TP	0.0078 (0.59)	−0.0010 (−0.66)	0.0043^***^ (3.46)	−0.0003 (−0.29)	0.0061 (0.51)	0.0051^***^ (4.45)
RE	0.0001 (0.02)	−0.0003 (−0.97)	0.0017^***^ (4.67)	0.0000 (−0.05)	−0.0004 (−0.09)	0.0005 (1.10)
AF	−0.0074 (−0.47)	0.0038^**^ (2.34)	0.0005 (0.51)	−0.0003 (−0.39)	−0.0104 (−0.72)	0.0004 (0.45)
GS	0.0093 (1.41)	−0.0021^**^ (−2.08)	−0.0023^**^ (−2.43)	0.0008 (1.17)	0.0129^**^ (2.20)	−0.0060^***^ (−4.61)
AR (2)	0.326	0.444	0.501	0.018	0.077	0.022
Sargan’s test	0.466	0.004	0.751	0.000	0.476	0.320

^*^, ^**^, ^***^ represent that it is significant on 0.1, 0.05, 0.001, and the value in parentheses is the *t* statistic, the same below.

The empirical analysis results show that there is an obvious positive correlation between agricultural insurance and rural revitalization, and with the improvement of agricultural insurance development level, China’s rural revitalization level will also increase. In the overall regression model, the regression coefficient of agricultural insurance development level is 0.1633, which is significant positive at the confidence level of 1%, that is, every 1 unit increase in the development level of agricultural insurance will drive China’s rural revitalization level to increase by 0.1633 units. The results fully demonstrate that agricultural insurance can bring relatively more positive effects to policymakers in promoting rural revitalization. It can be seen that vigorously promoting agricultural insurance into rural areas and continuously improving the coverage of agricultural insurance and its compensation rate can bring obvious positive benefits to China’s rural revitalization.

Furthermore, in order to explore the specific impact of agricultural insurance development level on the five dimensions of rural revitalization, this paper regresses the five dimensions of rural revitalization in turn, and the results are shown in Table 4. Among the total results, only the regression results of agricultural insurance to the dimension of social etiquette and civility did not pass the significance test, that is, the empirical results showed that civilization and education effect of agricultural insurance was not obvious. One possible explanation is that agricultural insurance has less money to spend on improving infrastructure in rural areas; At the same time, agricultural insurance is a special work carried out by professionals, this part of the knowledge is more professional, and the knowledge that residents understand and learn from it is very limited. These reasons limit the civilization and education effect of agricultural insurance. In addition, in view of the fact that social etiquette and civility covers a very wide range and there are many influencing factors, when only one factor of agricultural insurance is used to affect social etiquette and civility, its influence is difficult to show, especially the impact of agricultural insurance on social etiquette and civility is relatively indirect, and it is difficult to see the impact of agricultural insurance factors on social etiquette and civility in the short term, so the coefficient is not significant in the model. In terms of countermeasures, relevant strategies can be added to strengthen the positive role of agricultural insurance in promoting the construction of social etiquette and civility. Agricultural insurance has a significant positive effect on the pleasant living environments index at the confidence level of 1%, and the coefficient of the development level of agricultural insurance in the ecological livability model is 0.0209. In addition, in the model of thriving businesses, effective governance, and prosperity, the coefficients of the development level of agricultural insurance were 0.0152, 0.1164, and 0.0090, respectively, which were significant at the confidence level of 5%, indicating that agricultural insurance also has a certain role in thriving businesses, effective governance, and prosperity.

### 3.3 Regional heterogeneity analysis

In order to further explore the impact of agricultural insurance on various regions in China, this paper divides Chinese provinces into three regions: eastern, central and western regions for regional heterogeneity testing. The eastern region includes 12 provinces, autonomous regions and municipalities directly under the central government including Beijing, Tianjin, Hebei, Liaoning, Shanghai, Jiangsu, Zhejiang, Fujian, Shandong, Guangdong, Guangxi and Hainan; The central region includes 9 provinces and autonomous regions including Shanxi, Inner Mongolia, Jilin, Heilongjiang, Anhui, Jiangxi, Henan, Hubei and Hunan; The western region includes 10 provinces and autonomous regions including Chongqing, Sichuan, Guizhou, Yunnan, Tibet, Shaanxi, Gansu, Ningxia, Qinghai and Xinjiang.

The results of the regional heterogeneity test are shown in Table 5. By comparing the three regions, it can be found that in the regression model of the eastern region, the coefficient of agricultural insurance development level is 0.0800, which is significant at the confidence level of 5%, while the coefficient of agricultural insurance development level is not significant in the central and western regional models.

**Table 5:** Results of the regional heterogeneity test.

Explanatory variable	RR	Eastern part	Central part	Western part
	(1)	(7)	(8)	(9)
L.RR	0.1632*	0.1942	0.3184*	0.2114
	(1.73)	(1.29)	(2.00)	(0.97)
AI	0.1633***	0.0800**	0.0757	0.2744
	(2.74)	(2.79)	(0.54)	(1.32)
PD	0.0782***	0.0718**	0.0771	−0.0221
	(3.06)	(2.28)	(0.78)	(−0.25)
MF	−0.0115**	−0.0233**	−0.0211**	0.0031
	(−2.08)	(−3.04)	(−3.22)	(0.22)
TP	0.0078	0.0204	0.019	0.0559
	(0.59)	(1.45)	(0.71)	(1.27)
RE	0.0001	−0.0064	−0.003	−0.0048
	(0.02)	(−0.93)	(−0.68)	(−0.59)
AF	−0.0074	0.0048	0.035*	−0.0759
	(−0.47)	(0.92)	(2.17)	(−1.64)
GS	0.0093	−0.0022	−0.020	0.0066
	(1.41)	(−0.20)	(−2.43)	(0.39)
AR (2)	0.326	0.421	0.072	0.718
Sargan’s test	0.466	0.740	0.745	0.193

^*^, ^**^, ^***^ represent that it is significant on 0.1, 0.05, 0.001, and the value in parentheses is the *t* statistic, the same below.

There are three possible reasons for the above differences: First, the difference in agricultural insurance capital input. There are differences in the level of economic development in the eastern, central and western regions, and the development of agricultural insurance is different, and the financial investment is different. The low level of economic development in the central and western regions, insufficient government financial funds, the development of agricultural insurance is relatively lagging behind, and relatively small funds for agricultural operators to purchase agricultural insurance hinder the role of agricultural insurance. The eastern region has a relatively high level of economic development, the development of agricultural insurance is relatively good, relatively large financial support, relatively rich farmers, and relatively more funds used by agricultural operators to purchase agricultural insurance, so the role of agricultural insurance in promoting rural revitalization is relatively more obvious. Second, the difference in agricultural insurance channels and agricultural insurance infrastructure construction. The eastern region, including the capital and two municipalities directly under the central government, as well as more coastal open cities, has relatively smoother agricultural insurance channels, more complete agricultural insurance supporting facilities, and a more complete agricultural insurance system. However, due to the narrow channels of agricultural insurance in the central and western regions, the policy objectives and orientation of agricultural insurance are vague, and the corresponding agricultural insurance financial subsidies cannot be matched, and there is no way to obtain central financial subsidies, and their promotion role is weak. Third, differences in farmers’ concepts. The different levels of economic development in the eastern, central and western regions have also led to differences in the concepts of farmers. In the eastern region, where the economic level is higher, farmers are more aware of relying on market mechanisms to resist and diversify risks, and are more willing to buy agricultural insurance. However, most farmers in the central and western regions regard agricultural insurance as their own economic burden, which inhibits the development of local agricultural insurance.

### 3.4 Threshold effect analysis

#### 3.4.1 Construction of threshold effect model

The main function of the systematic GMM model is to judge the effect of agricultural insurance on rural revitalization and its five dimensions, but it cannot explain whether the effect of agricultural insurance on rural revitalization is linear or has a threshold effect. The threshold effect refers to the effect that when the relevant economic variable reaches a certain threshold value, the parameters of another economic variable will change, and a structural mutation will occur. The threshold effect model is an estimation method that further verifies the nonlinear relationship between the dependent variable and the explanatory variable based on the threshold value in the dynamic panel data. In order to solve this problem, this paper selects the proportion of agricultural insurance scale as the threshold variable, and explores the threshold range in which the proportion of agricultural insurance can effectively improve the level of rural revitalization. The threshold effect model is as follows:


(11) $$\begin{array}{c} Y_{it}=\alpha_{1}+\alpha_{2}\;AI_{it}\left(\alpha i_{it}<\beta_{1}\right)+\alpha_{3}\;AI_{it}\left(\beta_{1}⩽\right.\left.\alpha i_{it}⩽\beta_{2}\right)\\ +\alpha_{4}\;AI_{it}\left(\beta_{2}<\alpha i_{it}\right)+\alpha_{5}X+\varepsilon_{it} \end{array}$$

In Formula (11), $Y_{it}$ is the explanatory variable, representing the overall development level of rural revitalization and its five dimensions; $AI_{it}$ is the core explanatory variable, indicating the level of agricultural insurance development; The threshold scalar is the proportion of agricultural insurance scale (expressed by $\alpha i_{it}\;$ to reflect the income and expenditure of agricultural insurance), $\beta_{1}$ is the first threshold, $\beta_{2}\;$ is the second threshold, and $\alpha_{1},\;\;\alpha_{2},\;\;\alpha_{3},\;\;\alpha_{4}\;$ and $\alpha_{5}$ are all parameters to be estimated; X are the other control variables; $\varepsilon_{it}$ is a random perturbation term; The subscript i indicates each region and t indicates the year.

#### 3.4.2 Empirical research

In order to ensure the precision of threshold estimation, the Hansen bootstrapping method was used to determine the threshold number of the model, and the single-threshold, double-threshold and triple threshold tests were carried out in turn, and the test results are shown in Table 6. The test results show that only the double threshold effect is significant at the confidence level of 1%, and the impact of agricultural insurance on rural revitalization is not a simple linear relationship, and there is a significant double threshold feature between the two.

**Table 6:** Threshold effect test results.

Threshold variables	*F*-value	*p*-value	Number of samples	Threshold value		
				1%	5%	10%
Single threshold	11.94	0.148	500	13.3861	17.0314	24.533
Double threshold	50.75^***^	0.000	500	16.9791	21.3588	29.3289
Triple threshold	7.96	0.678	500	135.1157	170.7016	245.9360

^***^represent that it is significant on 0.001, and the value in parentheses is the *t* statistic, the same below.

After passing the threshold effect test, this paper analyses the double threshold estimate. Table 7 shows the estimated values of the threshold and the corresponding confidence intervals, the estimated values of the thresholds are 0.0638 and 0.0651, respectively, according to which the proportion of agricultural insurance can be divided into three intervals from low to high. On this basis, the nonlinear double threshold model of agricultural insurance scale proportion on rural revitalization is further regression, and the results are shown in Table 8. The results show that the positive impact of agricultural insurance on rural revitalization has a moderately optimal level, and the fluctuation effect of the development scale of agricultural insurance is an important reason for the existence of the double threshold effect of agricultural insurance to promote rural revitalization.

**Table 7:** Results of the double threshold estimation.

Threshold variables	Estimated threshold	95% Confidence interval
γ1	0.0638	[0.0585, 0.0651]
γ2	0.0651	[0.0638, 0.0668]

**Table 8:** Estimated results of the double-threshold regression model.

Variables	Coefficient estimate	Standard error	*T*-value
L.RR	−0.137^***^	0.049	−2.82
PD	0.138^***^	0.023	5.89
MF	−0.009^**^	0.004	−2.07
TP	0.007	0.011	0.67
RE	−0.004	0.003	−1.26
AF	−0.006	0.011	−0.50
GS	0.003	0.008	0.44
Percentage of agricultural insurance size ≤ 0.0638	0.020	0.028	0.70
0.0638 ≤ Percentage of agricultural insurance size ≤ 0.0651	0.497^**^	0.263	1.89
Percentage of agricultural insurance size ≥ 0.0651	0.039	0.031	1.26
constant	−0.890^***^	0.218	−4.09

^**^, ^***^ represent that it is significant on 0.05, 0.001, and the value in parentheses is the *t* statistic, the same below.

#### 3.4.3 Analysis of results

The regression results show that there are significant differences in the impact of agricultural insurance on rural revitalization under different agricultural insurance scale proportions. When the proportion of agricultural insurance is lower than the estimated value of the first threshold or higher than the estimated value of the second threshold, the impact of agricultural insurance on rural revitalization is not significant; When the proportion of agricultural insurance scale is between the estimated value of the first threshold and the estimated value of the second threshold, the estimated value of the proportion coefficient of agricultural insurance scale increases sharply and is significant at the level of 1%, and agricultural insurance shows a significant positive impact on rural revitalization.

The characteristics of the double-threshold effect of agricultural insurance on rural revitalization show that in the first stage, when the proportion of agricultural insurance is lower than the first threshold, the development of agricultural insurance is limited, and the amount of funds of small-scale agricultural insurance is insufficient to effectively improve the level of rural revitalization. In the second stage, when the proportion of agricultural insurance is higher than the first threshold and lower than the second threshold, with the gradual expansion of the proportion of agricultural insurance, agricultural insurance can effectively improve the level of rural revitalization. In the third stage, when the proportion of agricultural insurance is higher than the second threshold, the impact of agricultural insurance on rural revitalization is no longer significant. The main reasons may be: the disposable income of agricultural producers is limited, and the proportion of agricultural insurance is too large, which will lead to insufficient investment in other fields, thereby inhibiting the improvement of rural revitalization; The main function of agricultural insurance is to disperse the risks that may occur in the future, and the excessive proportion of agricultural insurance will make farmers invest too much in agricultural insurance, resulting in a decrease in the cost performance of agricultural insurance and breeding fluke psychology, which will make some agricultural operators choose to abandon the purchase of agricultural insurance, thereby restricting the impact of agricultural insurance on rural revitalization.

## 4 Conclusion and policy suggestions

### 4.1 Research conclusion

Through the TOPSIS entropy weight method, this paper finds that the actual development level of rural revitalization is not completely consistent with the level of economic development. Through the system generalized method of moments (GMM), it is found that agricultural insurance has a significant role in promoting the development level of rural revitalization, but the promotion effect of agricultural insurance on the five goals of rural revitalization is different. At the same time, the effect of agricultural insurance on the level of rural revitalization in different regions is quite different. The role of agricultural insurance in rural revitalization has a double threshold effect. The details are as follows:

1. An empirical study on the level of rural revitalization in various provinces in China through the TOPSIS entropy weight method. The empirical results show that the level of rural revitalization in various provinces in China shows a dynamic trend of “overall slow rise, with obvious differences between provinces.” Only three provinces, Guangdong, Henan and Jiangsu, improved their rural revitalization level by more than 0.1. The level of rural revitalization in Shanxi Province and Tianjin City showed negative growth. A more significant finding is that the level of economic development can only partially measure the results of rural revitalization and development, while rural revitalization is an agricultural and rural modernization that encompasses five dimensions and requires comprehensive development.
2. The system generalized method of moments (GMM) method was used to empirically study the effect of agricultural insurance on the level of rural revitalization in various provinces of China and its five dimensions. The empirical results show that with the improvement of the development level of agricultural insurance, the level of rural revitalization also increases, and every 1 unit increase in the development level of agricultural insurance will drive the level of rural revitalization in China to increase by 0.1633 units. Specifically, every 1 unit increase in the development level of agricultural insurance will increase the thriving businesses of 0.0152 units, the pleasant living environments of 0.0209 units, the effective governance of 0.1164 units and the prosperity of 0.0090 units. However, the regression results of agricultural insurance to the dimension of social etiquette and civility did not pass the significance test. In general, vigorously promoting agricultural insurance into rural areas can bring positive benefits to China’s rural revitalization.
3. The system generalized method of moments (GMM) method was used again to conduct an empirical study on the regional heterogeneity of the effect of agricultural insurance. Empirical results show that the impact of agricultural insurance on the level of rural revitalization in different provinces in China varies according to the region. The coefficient of the development level of agricultural insurance in the eastern region is 0.0800, which is significant at the confidence level of 5%. The effect of agricultural insurance on rural revitalization is not significant in the central region and the western region. The main reasons for regional heterogeneity may be the differences in capital input for agricultural insurance development, the differences in agricultural insurance channels and agricultural insurance infrastructure construction, and the differences in farmers’ concepts.
4. A threshold effect model was constructed to empirically study whether the impact of agricultural insurance on rural revitalization had a threshold effect. The proportion of agricultural insurance scale was selected as the threshold variable for empirical testing, and the empirical results showed that the impact of agricultural insurance on rural revitalization had a double threshold effect. The impact of agricultural insurance on rural revitalization is significantly different due to the proportion of agricultural insurance scale: when the proportion of agricultural insurance scale is lower than the estimated value of the first threshold or higher than the estimated value of the second threshold, the impact of agricultural insurance on rural revitalization is not significant; When the proportion of agricultural insurance is between the estimate of the first threshold and the estimate of the second threshold, agricultural insurance begins to show a significant role in promoting rural revitalization.

### 4.2 Policy suggestions

According to the research conclusions of this paper, the following policy suggestions for the development of agricultural insurance to promote rural revitalization are put forward:

1. Formulate agricultural insurance support policies and increase capital investment in agricultural insurance. On the whole, increasing agricultural insurance input can drive the improvement of rural revitalization, so governments at all levels and their relevant departments should increase financial support for agricultural insurance policies, cooperate with relevant incentive policy support, according to the different development stages of rural revitalization in each province, to meet the corresponding diversified needs, and effectively ensure the long-term stability of agricultural insurance support policies in various provinces.
2. Innovate a new mode of agricultural insurance operation and make up for the shortcomings of agricultural insurance. Specifically, it is mainly reflected in the following two aspects. First, guide agricultural insurance to focus on agricultural and rural infrastructure, encourage agricultural insurance to invest in agriculture and rural infrastructure construction, and leverage more medium and long-term credit funds into agricultural production and rural areas. At the same time, with the help of government-led, industry-coordinated, and insurance companies to promote the popularization of local cultural knowledge. Secondly, the agricultural insurance authorities and insurance companies jointly organize special training activities, while enhancing the publicity of agricultural insurance knowledge, appropriately cultivate a group of compound professional personnel who are familiar with agricultural insurance knowledge and understand agricultural production technology, so as to ensure that insurance companies can smoothly provide agricultural insurance services in rural areas, and at the same time, they can also provide a series of agricultural technical guidance and training to agricultural producers to help them improve their production technology and management level.
3. Based on the actual situation of rural areas in various places, promote the in-depth development of agricultural insurance in accordance with local conditions. Due to the regional heterogeneity of the impact of agricultural insurance on the level of rural revitalization, the effect in the central and western regions is not significant at present, so a single agricultural insurance policy cannot be adopted, and characteristic agricultural insurance should be developed according to the actual situation of rural areas in various places. Agricultural insurance is a kind of commercial activity, especially in the central and western regions to strengthen the construction of agricultural insurance infrastructure, based on the local additional agricultural insurance subsidies to increase the local commercial insurance incentive policy. Through a series of specific and differentiated characteristic agricultural insurance measures, regional heterogeneity of the role of agricultural insurance is eliminated to the greatest extent.
4. Pay attention to the double threshold effect of agricultural insurance and reasonably adjust the capital input of agricultural insurance. Considering that the proportion of agricultural insurance in a reasonable range can it play a positive role and effectively promote rural revitalization and development. In 2020, China became the country with the largest agricultural insurance premiums for the first time, but it still presents the characteristics of “large but not refined,” and the central government’s subsidies for local special agricultural products are limited to the types of agricultural insurance that are included in the “substitution of awards for subsidies,” and the scope and scale of subsidies are limited. Therefore, in order to maximize the development level of rural revitalization, the government and the market need to work together to promote, and enhance the positive role of agricultural insurance in the process of rural revitalization, the scale of agricultural insurance should be more accurately approved, and the amount of agricultural insurance should be appropriately disbursed. This requires agricultural insurance authorities and insurance companies to reasonably plan premium standards, so that agricultural insurance can play a positive role in agricultural insurance while not increasing the burden on farmers.

In summary, this paper deeply analyzes the impact of agricultural insurance on rural revitalization and its mechanism through the TOPSIS entropy weight method, the system generalized method of moments (GMM) method and the threshold effect model, and finds that the level of rural revitalization in various provinces in China is generally not high, showing a dynamic trend of “overall slow increase, obvious provincial differences,” and the promotion effect of agricultural insurance on the level of rural revitalization and its five dimensions in China’s provinces is not the same, and there are also differences between regions, and there is a double threshold effect. In view of this, when using agricultural insurance to promote rural revitalization, it is not possible to adopt a simple strategy, but should combine local characteristic resources and practical needs according to local conditions, so that it can fully serve the overall goal of rural revitalization.

## 5 Research deficiencies and prospects

This article still has some shortcomings. For example, statistics are short-lived. It has been nearly 20 years since the Central Document No. 1 first proposed agricultural insurance in 2004. However, in order to study the heterogeneity of various regions, and limited by the availability of data, this paper only starts from 2010 when the central government subsidizes the heterogeneity of various regions, and only uses data from 2011 to 2020, which does not fully cover the entire development process of agricultural insurance to the present. In addition, this paper mainly conducts research from a macro perspective, lack of research on the actual situation, and does not pay attention to the influence of rural micro-subjects on the role of agricultural insurance on rural revitalization.

In the future, the coverage of statistical data can be expanded to comprehensively monitor the dynamic trend of the impact of agricultural insurance on rural revitalization. At the same time, the impact of agricultural insurance on households can be further explored through field research, questionnaires and interview analysis. In this way, the impact of agricultural insurance on rural revitalization can be analyzed from the perspective of household micro, which can be used as a future research direction.

## Data availability statement

The original contributions presented in the study are included in the article/supplementary material, further inquiries can be directed to the corresponding authors.

## Author contributions

CZ: Writing – review & editing. JL: Writing – review & editing. SW: Writing – review & editing. HZ: Writing – review & editing. SC: Writing – review & editing.

## Footnotes

^2^In 2019, the Ministry of Finance, the Ministry of Agriculture and Rural Affairs, the China Banking and Insurance Regulatory Commission and the Forestry and Grassland Administration jointly issued the “Guiding Opinions on Accelerating the High-quality Development of Agricultural Insurance,” which put forward new positioning and new requirements for agricultural insurance in the new stage and new era to promote the development of modern agriculture and promote the revitalization of rural industries.
